# Supplementary material for: Phenotyping of Potato Plants Using Morphological and Physiological Tools
Source: Plants (Basel). 2024 Feb 26;13(5):647. doi: 10.3390/plants13050647 (PMC10934635; doi:10.3390/plants13050647)
Supplement: Supplementary file 1 [file plants-13-00647-s001.zip › plants-2809326-supplementary.pdf]

Table S1. Tuber Yield values of potato varieties for 2022–2023.

Variety	Numberof tubers per plant, pcs.			Average weight of tubers, g			Productivity, t/h		
	**2022**	**2023**	**Cv**	**2022**	**2023**	**Cv**	**2022**	**2023**	**Cv**
Early-ripening									
Argo	30.5	9.2	**76.1**	31.1	68.0	**52.6**	45.2	29.5	**29.8**
Bashkirsky	7.5	5.5	**21.8**	46.7	110.9	**57.6**	16.7	29.1	**38.3**
Blossom	9.0	7.1	**16.8**	61.1	105.1	**37.4**	26.2	33.8	**17.9**
Gala (standard)	7.5	9.9	19.5	66.7	52.6	**16.7**	23.6	24.4	**2.4**
Krasnoyarsky ranniy	7.0	6.6	**4.2**	85.7	121.1	**24.2**	28.6	35.5	**15.2**
Kholmogorsky	8.5	5.6	**29.3**	76.5	80.3	**3.4**	31.0	21.4	**25.9**
Polyarny	6.0	8.4	**23.6**	50.0	59.5	**12.3**	14.3	23.8	**35.4**
Sinichka	13.0	5.0	**62.9**	65.4	85.5	**18.8**	40.5	19.3	**50.1**
Sprinter	5.0	5.6	**8.0**	50.0	60.2	**13.1**	11.9	16.1	**21.2**
Middle-early									
Ariel	8.0	8.0	**0.0**	121.8	72.8	**35.6**	46.4	26.5	**38.7**
Alka	9.0	8.5	**4.1**	116.7	94.1	**15.2**	49.9	39.3	**16.8**
Bagira	13.0	7.4	**38.8**	46.1	74.2	**33.1**	28.6	25.0	**9.5**
Dvinsky	14.0	10.1	**23.0**	39.3	73.5	**42.9**	26.2	35.4	**21.1**
Farn	10.0	9.0	**7.4**	65.0	61.1	**4.4**	30.9	25.8	**12.7**
Ilyinsky (standard).	16.0	9.9	**33.4**	50.0	73.1	**26.5**	38.1	33.0	**10.1**
Kaluzhsky	10.0	9.1	**6.6**	70.0	101.6	**26.2**	33.2	43.7	**19.3**
Orlan	14.0	8.2	**36.9**	50.0	79.8	**32.5**	33.2	30.3	**6.5**
Visa	6.5	8.7	**20.5**	69.2	83.1	**12.9**	21.6	34.2	**31.9**
Mid-season-ripening									
Chaika	9.0	6.0	**28.3**	50.0	69.6	**23.2**	21.4	21.1	**1.0**
Dalnevostochny	12.5	8.9	**23.8**	48.0	40.5	**12.0**	28.5	17.2	**35.0**
Evpatiy	9.0	9.2	**1.6**	69.4	81.0	**10.9**	29.8	35.1	**11.6**
Intelligent	5.5	6.9	**16.0**	81.8	95.4	**10.9**	21.4	30.9	**25.7**
Moryak	10.5	5.1	**49.0**	64.3	75.0	**10.9**	32.1	17.8	**40.6**
Princessa Natavan	5.5	5.0	**6.7**	81.8	50.5	**33.5**	21.4	11.5	**42.4**
Spiridon	9.5	6.9	**22.4**	94.7	70.6	**20.6**	42.7	23.2	**41.9**
Tarasov	7.5	8.0	**4.6**	133.3	88.3	**28.7**	47.6	33.9	**23.8**
Tersky	7.5	6.9	**5.9**	110	63.2	**38.2**	39.3	18.7	**50.2**
Tomichka	14.5	5.9	**59.6**	68.9	44.3	**30.7**	47.6	12.8	**81.5**
Zhigulevsky (standard)	16.5	6.4	**62.6**	54.5	82.4	**28.8**	42.8	28.8	**27.7**

Table S2. Duration of phenological phases for potato plants of various varieties during the growing seasons 2022–2023.

Variety	Phenological stages								
	Planting, full germination			Full germination, beginning of flowering			Flowering duration		
	2022	2023	Cv	2022	2023	Cv	2022	2023	Cv
Early-ripening									
Argo	23	22	**3.1**	25	24	**2.9**	11	19	**37.7**
Bashkirsky	22	20	**6.7**	27	19	**24.6**	18	14	**17.7**
Blossom	23	22	**3.1**	30	20	**28.3**	10	12	**12.9**
Gala (standard)	26.	22	**11.8**	27	21	**17.7**	18	6	**70.7**
Krasnoyarsky ranniy	23	22	**3.1**	24	20	**12.9**	10	10	**0.0**
Kholmogorsky	26	20	**18.4**	24	26	**5.7**	6	8	**20.2**
Polyarny	26	18	**25.7**	25	21	**12.3**	5	14	**67.0**
Sinichka	26	27	**2.7**	28	21	**20.2**	10	7	**25.0**
Sprinter	26	20	**18.4**	15	19	**16.6**	10	15	**28.3**
Middle-early									
Alka	23	20	**14.0**	19	20	**3.4**	15	15	**0**
Ariel	22	22	**0**	12	17	**24.4**	27	18	**28**
Bagira	26	22	**16.7**	14	17	**13.7**	23	12	**44**
Dvinsky	26	20	**26.1**	30	22	**21.8**	5	12	**58**
Farn	22	20	**9.5**	20	28	**19.5**	19	7	**65**
Ilyinsky (standard)	23	20	**14.0**	19	34	**40.0**	17	5	**77**
Kaluzhsky	22	20	**9.5**	25	26	**2.8**	10	11	**7**
Orlan	26	25	**3.9**	31	30	**2.3**	4	7	**39**
Visa	23	18	**24.4**	30	30	**0.0**	8	8	**0**
Mid-season-ripening									
Chaika	22	25	**9.0**	19	23	**0.0**	24	22	**6.1**
Dalnevostochny	22	25	**9.0**	30	31	**4.6**	15	9	**35.4**
Evpatiy	22	22	**0**	27	25	**13.6**	13	16	**14.6**
Intelligent	22	20	**6.7**	19	26	**6.3**	35	19	**41.9**
Moryak	22	20	**6.7**	24	26	**2.8**	4	11	**66.0**
Princessa Natavan	22	25	**9.0**	20	25	**6.3**	18	5	**79.9**
Tarasov	22	18	**14.1**	18	28	**6.1**	22	11	**47.1**
Tersky	25	25	**0**	14	14	**35.4**	38	29	**19.0**
Tomichka	25	28	**8.0**	21	23	**12.9**	10	3	**76.1**
Spiridon	21	22	**3.3**	18	20	**37.2**	19	6	**73.5**
Zhigulevsky	25	22	**9.0**			**8.2**			**44.4**

Table S3. Field virus resistance of potato leaves

Variety	Virus resistance											
	X			Y			L			S		
	2022	2023	Means	2022	2023	Means	2022	2023	Means	2022	2023	Means
Early-ripening												
Argo	9.0	8.0	**8.5**	9.0	8.5	**8.8**	9.0	9.0	**9.0**	9.0	7.5	**8.3**
Bashkirsky	8.0	8.0	**8.0**	8.0	8.5	**8.8**	9.0	9.0	**9.0**	9.0	6.2	**7.6**
Blossom	9.0	7.7	**8.4**	9.0	3.0	**6.0**	9.0	9.0	**9.0**	9.0	3.0	**6.0**
Gala (standard)	8.0	8.7	**8.4**	9.0	9.7	**7.9**	9.0	9.0	**9.0**	8.0	4.7	**6.4**
Polyarny	7.0	8.0	**7.5**	9.0	8.0	**8.5**	9.0	9.0	**9.0**	6.0	4.5	**5.3**
Krasnoyarsky ranniy	9.0	8.7	**8.9**	9.0	8.5	**8.8**	9.0	9.0	**9.0**	9.0	6.7	**7.9**
Kholmogorsky	7.0	7.5	**7.3**	6.0	5.7	**5.9**	9.0	9.0	**9.0**	9.0	6.0	**7.5**
Sinichka	9.0	6.7	**7.9**	9.0	9.0	**7.5**	9.0	9.0	**9.0**	9.0	5.0	**7.0**
Sprinter	5.0	6.2	**5.6**	4.0	7.5	**5.8**	9.0	9.0	**9.0**	5.0	5.2	**5.1**
Middle-early												
Alka	9.0	9.0	**9.0**	9.0	9.0	**9.0**	9.0	9.0	**9.0**	9.0	8.5	**8.8**
Ariel	9.0	9.0	**9.0**	9.0	9.0	**9.0**	9.0	9.0	**9.0**	9.0	8.2	**8.6**
Bagira	9.0	9.0	**9.0**	9.0	8.2	**8.6**	9.0	9.0	**9.0**	7.0	5.0	**6.0**
Dvinsky	9.0	8.5	**8.8**	9.0	8.0	**8.5**	9.0	9.0	**9.0**	9.0	7.0	**8.0**
Farn	9.0	9.0	**9.0**	9.0	4.0	**6.5**	9.0	9.0	**9.0**	9.0	6.5	**7.8**
Ilyinsky (standard)	9.0	8.2	**8.6**	9.0	6.5	**7.8**	9.0	9.0	**9.0**	9.0	7.7	**8.4**
Kaluzhsky	9.0	8.2	**8.6**	9.0	7.0	**8.0**	9.0	9.0	**9.0**	8.0	8.0	**8.0**
Orlan	9.0	9.0	**9.0**	9.0	9.0	**9.0**	9.0	9.0	**9.0**	6.1	6.1	**6.1**
Visa	9.0	5.5	**7.3**	9.0	8.2	**8.6**	9.0	9.0	**9.0**	9.0	6.2	**7.6**
Mid-season-ripening												
Chaika	7.0	6.7	**6.9**	9.0	7.2	**8.1**	9.0	9.0	**9.0**	7.0	6.7	**6.85**
Dalnevostochny	8.3	8.5	**8.4**	8.6	7.2	**7.9**	9.0	9.0	**9.0**	8.1	7.1	**7.6**
Evpatiy	9.0	9.0	**9.0**	9.0	9.0	**9.0**	9.0	9.0	**9.0**	9.0	9.0	**9.0**
Intelligent	9.0	9.0	**9.0**	9.0	9.0	**9.0**	9.0	9.0	**9.0**	9.0	7.0	**8.0**
Moryak	9.0	9.0	**9.0**	9.0	9.0	**9.0**	9.0	9.0	**9.0**	9.0	9.0	**9.0**
Princessa Natavan	8.0	8.0	**8.0**	8.0	7.0	**7.5**	9.0	9.0	**-**	7.0	9.0	**8.0**
Spiridon	9.0	9.0	**9.0**	9.0	7.0	**8.0**	9.0	9.0	**90.**	9.0	6.2	**7.6**
Tarasov	8.0	7.7	**7.9**	9.0	3.0	**6.0**	9.0	9.0	**9.0**	9.0	7.7	**8.35**

Tomichka	7.0	9.0	**8.0**	8.0	8.0	**8.0**	9.0	9.0	**9.0**	5.0	3.0	**4.0**
Zhigulevsky (standard)	9.0	8.7	**8.9**	7.0	5.7	**6.35**	9.0	9.0	**9.0**	9.0	6.2	**7.6**
